# An Acoustic Camera for Use on UAVs

**DOI:** 10.3390/s23020880

**Published:** 2023-01-12

**Authors:** Iva Salom, Goran Dimić, Vladimir Čelebić, Marko Spasenović, Milica Raičković, Mirjana Mihajlović, Dejan Todorović

**Affiliations:** 1 Institute Mihailo Pupin, University of Belgrade, Volgina 15, 11060 Belgrade, Serbia; 2 Center for Microelectronic Technologies, Institute of Chemistry, Technology and Metallurgy, University of Belgrade, Njegoševa 12, 11000 Belgrade, Serbia; 3 Dirigent Acoustics, Mažuranićeva 29, 11000 Belgrade, Serbia

**Keywords:** acoustic camera, unmanned aerial vehicles, noise monitoring, border surveillance, wildlife monitoring, rescue missions, FPGA SoC

## Abstract

Airborne acoustic surveillance would enable and ease several applications, including security surveillance, urban and industrial noise monitoring, rescue missions, and wildlife monitoring. Airborne surveillance with an acoustic camera mounted on an airship would provide the deployment flexibility and utility required by these applications. Nevertheless, and problematically for these applications, there is not a single acoustic camera mounted on an airship yet. We make significant advances towards solving this problem by designing and constructing an acoustic camera for direct mounting on the hull of a UAV airship. The camera consists of 64 microphones, a central processing unit, and software for data acquisition and processing dedicatedly developed for far-field low-level acoustic signal detection. We demonstrate a large-aperture mock-up camera operation on the ground, although all preparations have been made to integrate the camera onto an airship. The camera has an aperture of 2 m and has been designed for surveillance from a height up to 300 m, with a spatial resolution of 12 m.

## 1. Introduction

Acoustic environmental noise pollution is becoming a noticeable issue, especially in urban or industrial environments. Regulatory bodies worldwide have prescribed noise pollution mapping and mitigation directives. Apart from the regulation-driven needs, noise pollution surveying has large potential on the wider surveying and Smart City markets, for use in traffic monitoring, automated route guidance, industrial noise monitoring, and environmental research.

Already in 1999, 65% of the European Common Market population was exposed to unhealthy levels of transportation noise [1]. Since those times, regulatory agencies across the globe have been setting noise level targets. In the EU, noise pollution is governed by the Environmental Noise Directive—END (Directive 2002/49/EC). END requires Member States to monitor noise and prepare and publish noise maps for cities, industry, and transportation connections. Strategic noise mapping is currently performed by inputting noise source models, terrain configuration, objects, and sound propagation conditions into numerical simulations that generate a prediction of average noise levels on the facades of residential buildings. However, current noise simulation methods have shown standard deviations of over 1 dB [2], which may cause overuse or underuse of protective measures.

Similarly, in the case of industrial noise and, in the last decade, drone noise, standard measurement techniques [3] often fail to register noise sources or give incorrect data. For more detailed analysis of industrial noise, experts utilize stationary acoustic sensors and cameras; however, some noise sources practically cannot be measured from the ground, especially when there are several sources of significant noise on one structure. In addition, such measurements give no information on noise source directionality, which is an essential parameter for applying adequate noise mitigation measures. Acoustic noise surveying from the air would enable detailed mapping of all noise sources, their sound power, frequency spectrum and directivity, and easy integration into geographic information systems (GIS). Aside from use in noise pollution, an acoustic camera mounted on an airship would be of use in rescue missions or surveillance of long, uninhabited borders for illegal border crossing. Illegal border crossing is an increasing problem, particularly felt in Europe as migratory pressures rise. According to Frontex, the total number of illegal border crossings in 2021 was near 200,000 [4]. These numbers are on the rise again, headed towards the pre-pandemic records of more than half a million illegal crossings per year.

Finally, airborne acoustic surveillance can be used for wildlife monitoring and research. Wildlife monitoring is important for species conservation, as researchers track animal feeding, mating, and migration patterns. Wildlife acoustic monitoring is increasingly being performed with meshes of acoustic sensors attached to trees in forests, but such meshes have limited resolution, require much human effort to install, and rely on complex data collection and labor-intensive record processing [5]. Acoustic surveillance from an airship would allow deployment to any region, even hard-to-reach geographies, dynamic monitoring even where fixed sensors are not installed, automated data collection, and rapid real-time surveillance.

Regardless of all the mentioned potential advantages, no acoustic camera to date has been designed for use on an airship and for aerial surveillance. Due to the typically large size of airships, it would be possible to construct large-aperture airborne acoustic cameras, which would yield high acoustic resolution, especially at lower frequencies, which leads to increased fidelity compared to cameras with smaller apertures that were designed for handheld applications.

An acoustic camera (AC) is an imaging device used to locate sound sources and to characterize them. It consists of a group of microphones, also called a microphone array, from which signals are simultaneously collected and processed to form a representation of the angular location of the sound sources with respect to the position of the AC. Visualization of the sound field is performed by applying a certain algorithm for spatio-temporal signal processing from the microphone array, creating an acoustic map that may be overlayed with a recording from a co-located video camera. Since the first AC appeared on the market at the beginning of the 2000s, the rapid development of this technology has started, so that today the acoustic camera is a modern engineering tool that, through the identification and determination of the position of sound sources, as well as the quantification and analysis of individual sound sources, is increasingly used for different purposes: for determining and characterizing noise sources, in the analysis of room acoustics, for testing sound insulation, for determining faults in industrial plants (detection/monitoring of sound field disturbances), when designing and testing vehicles (auto industry and aviation industry), in robotic systems, etc. [6,7,8,9,10,11,12,13]. As a consequence, a large number of ACs of different characteristics and realizations are available on the market today, depending on the application. The microphone arrays of ACs are controlled by a central processing unit (CPU). For custom-made cameras, the CPU can be implemented on a field-programmable gate array (FPGA) [7,14,15,16,17,18]. Several ACs that make use of FPGAs are in development, including ones with control algorithms optimized with gene sequencing methods [19]. Much work has been devoted to optimizing control algorithms where the algorithm is implemented on a personal computer [20]. Nevertheless, ACs designed specifically for mounting on an airship for airborne surveillance, in particular modern airships that may carry a multisensor payload, are lacking in the literature and on the market. Put succinctly, although all the necessary components for an AC mounted on an airship have recently reached maturity (FPGA-based ACs and powerful lightweight algorithms), there has been no coherent attempt to carefully select components and construct an airborne AC.

We made an AC dedicated for use on UAVs. The components of the AC attach directly to the frame of an airship, allowing for an aperture of several meters. The microphone boards and configuration are optimized for acquiring sound from terrestrial areas of up to 300 m × 300 m from an altitude of up to 300 m. The proposed solution can significantly speed up noise map making and increase map authenticity due to the inclusion of environmental acoustic noise sources. Furthermore, our aerial AC opens up the possibility of airborne acoustic surveillance for wildlife and security, including border security.

The AC consists of a microphone array and a data processing unit, implementing a beamforming algorithm to generate a visual presentation of the acoustic field (acoustic mapping) and two audio streams for detection and classification of the acoustic event. Microphone arrays are valuable tools for many applications, including specific sound-source detection, visualization, localization, and separation, also in a challenging acoustic environment, including indirect detection (via vegetation disturbance) [11,21].

Beamforming is a general signal processing technique used to control the directionality of the reception or transmission of a signal with an array of transducers. Acoustic beamforming is a signal processing technique used to enhance an audio system’s performance by focusing sound waves in a particular direction. It involves using an array of microphones to pick up sound from a particular source or direction and then using digital processing to combine the microphones’ signals to amplify the sound coming from the desired direction and suppress the noise coming from other directions. It can be used to determine the location (angular direction relative to the AC point of origin) and intensity of the sound source. Acoustic source localization is governed by the time difference in which the sound reaches the microphone array. As a measurement technique for sound-source location, beamforming is also useful at medium to long measurement distances, which is compatible with the described use cases.

**Figure 1:**
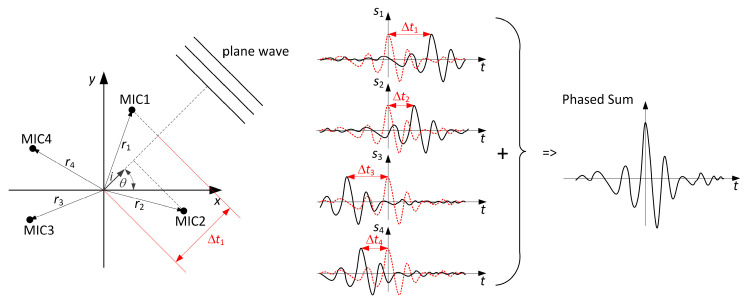
The conventional delay-and-sum algorithm in the time domain.

A commonly used algorithm is delay-and-sum (DAS) time domain beamforming [22]. The basic idea is presented in Figure 1. The sound source is assumed to be far from the microphone array (far field). Thus, the wavefront curvature approaches linearity relative to the size of the microphone array, and therefore it can be assumed that all incoming waves are plane waves.

There are many advantages of the DAS. The algorithm is based on basic concepts of wave propagation, such as linearity, straight-ray propagation, and weak back-scattering. The implementation of DAS is simple, and it can be parallelized. DAS is numerically robust, fast, and compatible with real-time applications, and because the algorithm is data-independent, it preserves temporal coherence and properties of the real envelopes [23].

DAS beamforming is implemented for testing and benchmarking of the AC array design performance metrics. For a finite distance between the sound source and the AC, derivation of the beamformer leads to cross-spectral imaging function. For adaptive beamforming, a minimum variance distortionless beamformer (MVDR) can be implemented [24].

## 2. Materials and Methods

The greatest challenge on the AC for sound-source localization from greater distances comes in the detection of low-level acoustic signals (up to 30 dB) due to sound propagation attenuation (−6 dB over each doubling of distance from the source) and absorption in air, especially at high frequencies. The AC was designed with this demand in mind.

Random noise (unwanted signal) suppression is achieved by selecting low self-noise microphones together with analogue and analogue-to-digital converter (ADC) electronic circuits, as well as with careful design of the windshield and EMI shielding implemented in the microphone and electronics case. Further improvement of the signal-to-noise ratio (SNR) is performed with a high-precision beamforming algorithm with 64 microphones. The SNR increases by 3 dB per doubling the number of microphones, yielding an SNR improvement of 18 dB for an array of 64 microphones. The audio signal is amplified and digitalized on the board next to each microphone to avoid interference. The useful signal frequency range is estimated to be approximately 200 Hz–2 kHz, whereas a sampling frequency of 8 kHz is chosen. This frequency spectrum is expected to carry most of the energy of acoustic events from the far field (on the ground). At higher frequencies, the detection precision increases until the wavelength approaches the spatial separation distance between the microphones (spatial sampling), which is dictated by the minimum distance between the microphones and the total camera dimensions. In addition, at high frequencies, the energy of acoustic events is expected to be low, as air attenuation is significant.

**Figure 2:**
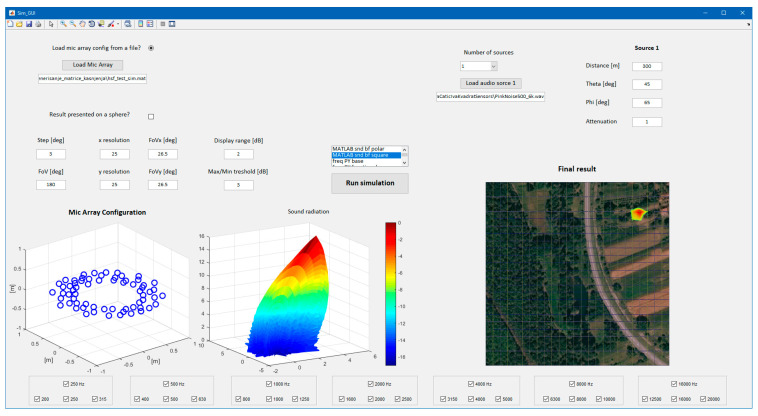
Simulation program interface.

A simulation was developed in MATLAB to investigate the behavior of the AC with different microphone configurations, utilizing different beamforming algorithms for a varying number, position, and characteristic of sound sources. Microphone positions were restricted to a geometry that is compatible with mounting the microphones on the frame of the airship (Hipersfera Border UAS, Zagreb, Croatia). A drawing of the airship frame with the position of the AC indicated is depicted in Supplementary Information Figure S1. The graphical user interface for the simulation is depicted in Figure 2, showing a simulation result for the implemented microphone configuration.

The microphone array is ring (annulus)-like. The largest distance between any two microphones is 2 m. The smallest distance between any two microphones is 0.09 m. The random spacing of the microphones keeps spatial aliasing and side lobe attenuation at an acceptable level for the desired frequency range.

The largest off-axis angle (angle of view/listen) is set to 26.5 degrees, which keeps the off-axis resolution at most 40% greater than the on-axis resolution. Maximum Sidelobe Level (MSL) is kept below −10 dB up to the wave number K_max_^26.5^. This fits well with the desired frequency range (200 Hz–2 kHz).

The microphones have a cardioid directionality pattern, with the largest gain in the direction perpendicular to the plane of the array. Within a cone near the main axis (+/−30°), the pattern can be approximated as omnidirectional.

**Figure 3:**
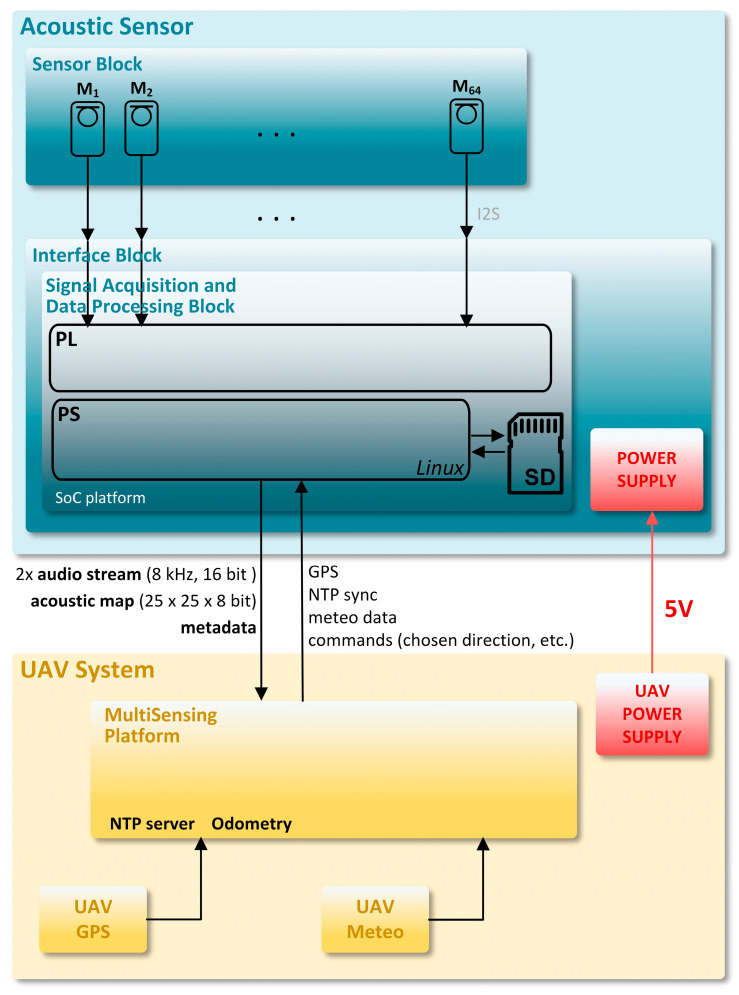
System architecture block diagram of the AC.

The system architecture block diagram of the AC is shown in Figure 3. The system consists of three blocks: a sensor block, an interface block, and a signal acquisition and data processing block.

The sensor block consists of a microphone array with 64 microphone modules. The microphone modules consist of microphones M1, M2, M3, …, M64 and analogue and digital circuits next to each microphone. Each microphone module performs microphone signal amplification (low-noise microphone preamplification), analog-to-digital conversion, and communication of the I2S digital audio signal (I2S bus specification, Philips Semiconductors, 1996) to the signal acquisition and data processing block via the interface block.

The interface block interfaces the microphone block to the signal acquisition and data processing block, as well as the signal acquisition and data processing block to the UAV. The UAV provides a 5 V DC power supply.

**Figure 4:**
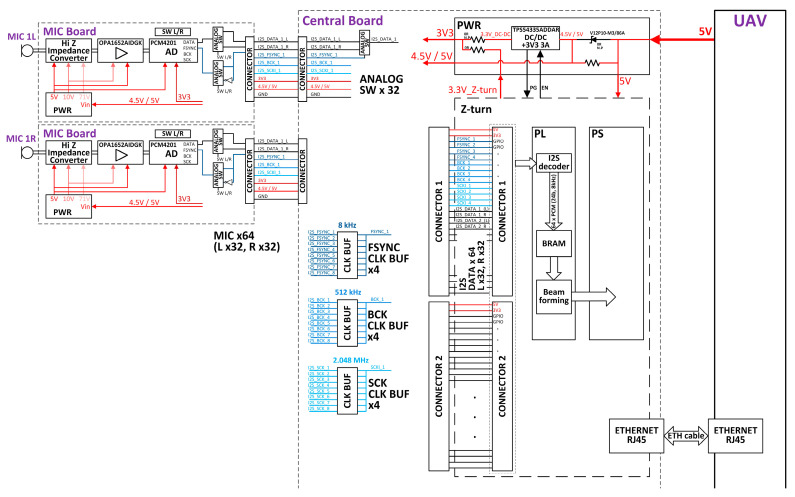
Hardware architecture of the acoustic camera.

The block diagram of the hardware architecture of the AC is presented in Figure 4 and includes 64 microphone boards/modules, a central board, and a Z-turn processing module (MYIR Tech Limited, Shenzhen, China).

Microphone modules were designed in-house, for optimal performance in the given use-case scenario. Required microphone specifications included low self-noise (<15 dBA), sensitivity greater than −33 dB/Pa (@48 V phantom power), a frequency range between 20 Hz and 20 kHz, and a cardioid directivity pattern. Commercially available microphone capsules were integrated onto custom-made electronics boards to create microphone modules. In order to increase the microphone sensitivity a polarization voltage of 71 V for the microphone capsule was applied.

A pair of microphone modules (“a left” and “a right”) are connected to the central board (the interface module) over a single 8-wire interface cable. The direction (left/right) of the microphone module is selected using a switch on the microphone module.

A microphone module consists of several blocks: power supply, high impedance converter, preamplifier and filters, AD converter and central board interface.

The power supply includes components for 5 V and 10 V generation—the power supplies for the components on the PCB, in addition to 3.3 V, as well as 71 V generation. The low self-noise preamplifier (<−130 dBV) with the corresponding filters provides a gain of 200.

The TI PCM4201 AD converter (Texas Instruments, Dallas, TX, USA) was chosen for this application for its low noise, low distortion, and low power, with supported sampling rates from 8 kHz to 108 kHz. The output of the AD converter is 24-bit linear left-justified PCM data, which can be considered as I2S.

Three clocks are generated on the signal acquisition and data processing block for the AD converter, as it operates in slave mode: a frame synchronization (FSYNC) clock for 8 kHz sampling, a bit (BCK) clock for 512 kHz sampling, and a system (SCK) clock for 2048 kHz sampling. These signals are transmitted over long 8-wire interface cables, where the maximal cable length is 2.4 m. For these frequencies and cable lengths, the signal integrity was checked in a simulation.

The switching between the left and right channel is performed at the FSYNC clock frequency. Power supplies for the microphone module (5 V and 3.3 V) are provided via the 8-wire interface cables as well.

The central board contains 32 headers for the microphone module interface, 3 × 4 clock buffers, 2 connectors for the Z-turn interface, and a power supply with the corresponding filters. The central processing unit of the system, used for signal acquisition and data processing, is the Z-turn board. The Z-turn board is a high-performance Single Board Computer (SBC) built around the Zynq-7020, as a member of the Xilinx All Programmable System-on-Chip (SoC) platform Zynq 7000 family, featuring integrated Programmable Logic (PL)—Xilinx 7-series Field-Programmable Gate Array (FPGA) logic and a Processing System (PS) consisting of dual-core ARM cortex A9 processors (Xilinx XC7Z010/020, San Jose, CL, USA).

**Figure 5:**
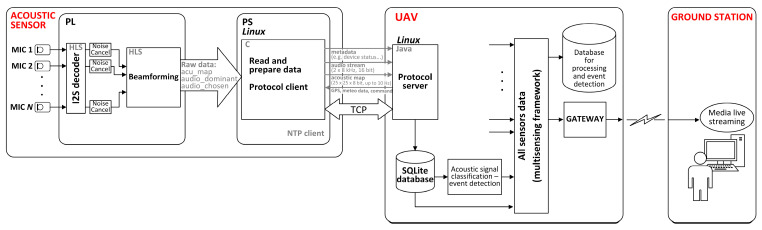
Software architecture of the AC.

**Figure 6:**
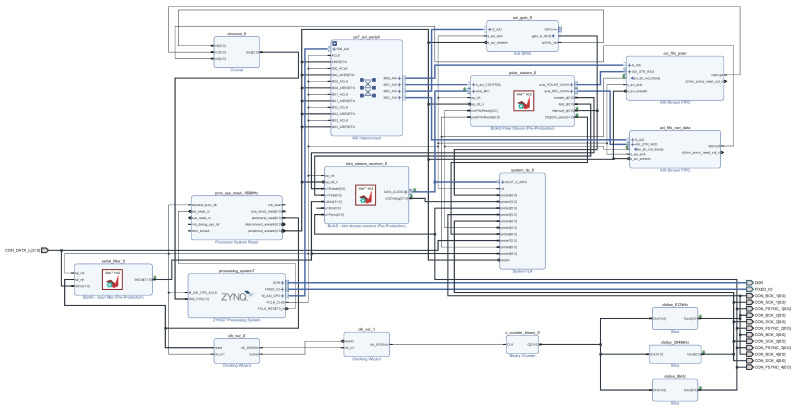
PL implementation in Xilinx Vivado Design Suite 2020.2.

The software architecture block diagram of the AC is depicted in Figure 5. The data acquisition and beamforming algorithm are implemented on PL, using the Vivado Design Suite 2020.2 environment (Xilinx, San Jose, CL, USA). Data acquisition is implemented in the tdm_stream_receiver Intellectual Property (IP) High-Level Synthesis (HLS) block, while the conventional delay and sum beamforming algorithm is implemented in the polar_stream IP HLS block, as presented in Figure 6.

The system operating frequency is 100 MHz. Data acquisition in the tdm stream receiver IP HLS block implies sampling of microphone signals, with a sampling frequency of 8 kHz (i.e., every 125 ms), and conversion into a serial data sequence. This block introduces a processing delay of 3 cycles of the operating frequency, i.e., 30 ns. The serial data sequence is forwarded to the polar _stream IP HLS block.

The outputs of the polar_stream IP HLS block are the following: a block of 625 32-bit integers containing values of each of the 25 × 25 fields of the acoustic map for each second (a memory block), and 24-bit audio samples from the dominant and a chosen direction, each 125 µs long (AXI-Stream FIFO). In addition, for verification purposes, a chosen microphone signal sample is sent to the PS every 10 ms (80 samples as a single packet in AXI-Stream FIFO).

The implementation of the conventional delay and sum beamforming algorithm in the polar_stream IP HLS block is optimized in terms of basic FPGA resources and processing time. A single pass through this block introduces a processing delay of 5 cycles of the operating frequency, i.e., 50 ns. The main steps within this IP HLS block are the following:

- Reading of serial audio data (from the tdm_stream_receiver IP HLS block) and packing these data into 64 shift registers: performed every 125 µs (the sampling frequency); the execution time is 50 ns × 64 = 3.2 µs.
- Calculation of the averaged polar matrix (25 × 25 fields of the acoustic map for each second) and sending the synchronization signal to PS: performed every 1 s; the execution time is 50 ns × (625 + 2) = 31.35 µs.
- Sending of 2 signals (the chosen and the dominant) to PS: performed every 125 µs; the execution time is 50 ns × 2 = 100 ns.
- Implementation of the conventional delay and sum beamforming algorithm: performed every 125 µs; the execution time is 50 ns × 625 = 31.25 µs.

The total required processing time, including all previously described steps, is 65.93 µs.

PS is based on the Linux Ubuntu 20.04 OS. A single Linux application performs data exchange with PL via two AXI-Stream FIFOs and a mapped shared memory, collecting raw data from PL in real time. The application generates three files each second: an acoustic map, which is a .bmp image file (25 × 25 pixels, 8-bit depth), and two .wav audio files (8 kHz, 16-bit depth), one for the dominant direction signal and another for a chosen sound-source signal. The application sends these files to the UAV via a communication protocol based on the User Datagram Protocol (UDP). The application also receives from the UAV meteorological data, GPS data, and commands. An NTP daemon service runs on the OS, whereas ntp.conf needs to be set according to the final UAV central system configuration (including NTP server settings).

The acoustic map is calculated using a beamforming algorithm-based delay matrix that depends on the microphone array configuration and meteorological data, especially wind speed, which affects sound speed, as well as the desired FoV (field of view). The delay matrix is calculated on the PS based on determined microphone positions and current meteorological status, and is sent to the PL.

The AC has a chosen FoV spanning ±26.5 degrees relative to the microphone array’s central axis. Thus, for an altitude of 300 m, the acoustic map will cover an area of 300 m × 300 m on the ground.

The AC should be connected to the UAV central unit via a single Ethernet connection, using the UDP protocol. The proprietary protocol is implemented for data exchange between the AC and the UAV. The acoustic map and two audio files are transferred to the UAV processing unit every second and stored with a filename containing the timestamp. The precise time on the central processing unit of the AC should be set using the NTP protocol, whereas the NTP server is implemented on the UAV processing unit. The UAV sends to the AC GPS coordinates, meteorological data, and control commands.

To summarize, both parallel data acquisition and beamforming are performed in PL, and signal processing results are transferred to the PS. The PS collects raw data from PL, generates .bmp and .wav files, and sends them to the UAV. The signal acquisition and data processing block are connected to the UAV via a single Ethernet connection. The protocol for the communication between the AC and the UAV is implemented on the PS using UDP. The communication is bidirectional, since the AC sends two audio streams, an acoustic map, and metadata, and receives GPS data with the precise time, meteorological data, and commands.

The operator (on the ground) can listen to the recorded audio signals. In addition to the sound coming from the dominant direction, the operator can choose another direction that they would like to monitor.

The AC system can be connected to a PC/laptop via a UART-to-USB connection for testing purposes.

The AC (microphone modules and central board) should ideally be mounted directly onto the frame of the UAV. In the case of a modern airship, which typically has a carbon tube internal construction [25], the AC is best mounted directly onto the tubes, because in such a case the AC would conform to the shape of the airship and there is no need for extra mounting that would increase the load. An optimized position for the AC components on a commercially available airship UAV, as confirmed with simulation, is shown in Supplementary Information Figure S1. For ground-testing purposes, we mounted the AC components on a circular piece of cardboard with a diameter of 2 m as a mock-up of actual operation on an airship.

The AC communicates with the UAV via our custom developed UDP Client-Server protocol. A test application for the server side (UAV) was written in JAVA and it can be run in Linux OS on a PC. The same application can be adapted for the final application on the UAV. The test application stores the received data (the acoustic maps and audio files) in an SQLite database, as well as files on the file system in the selected folders.

There are three tables in the SQLite database: acu_map, audio_dir_dominant, and audio_dir_chosen. Each second, a set of data (a .bmp and two .wav files) is stored as a BLOB field in the corresponding table of the database. The communication protocol defines the possible commands and responses (statuses), as well as the corresponding data (depending on the context) which are exchanged between the AC and the UAV. Each side receives command packets (CMD_*) and sends an appropriate response (RESP_*) for every received packet in the time interval specified for that command.

## 3. Results

### 3.1. Hardware Implementation

**Figure 7:**
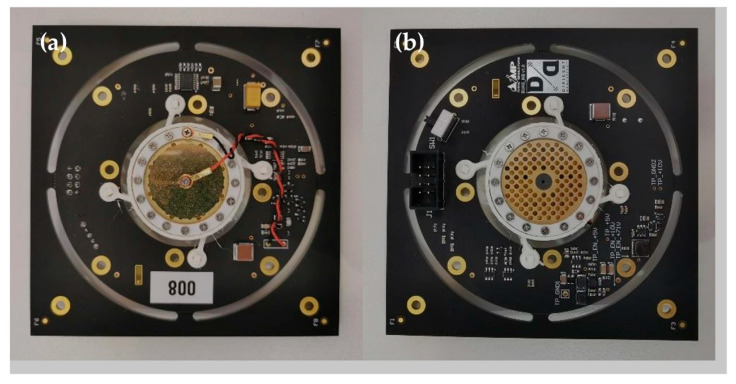
Assembled microphone module: (**a**) front view; (**b**) back view.

Photographs of an assembled microphone module front and back side are depicted in Figure 7. A total of 64 such modules are assembled and connected to the central board. Each microphone is protected with a wind shield and EMI shielding (see Supplementary Information Figure S2).

**Figure 8:**
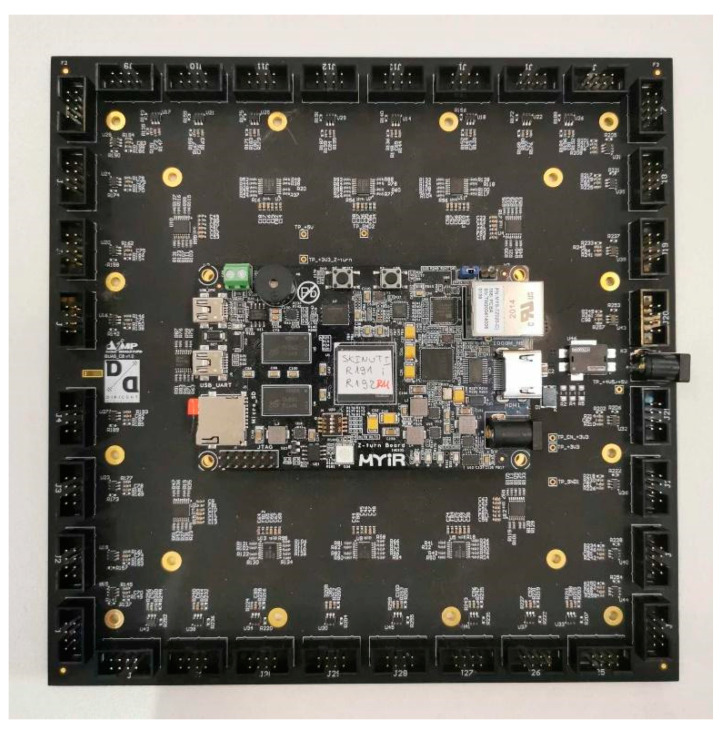
Assembled signal acquisition, data processing, and interface module.

A photograph of the assembled central board with the Z-turn board is depicted in Figure 8.

**Figure 9:**
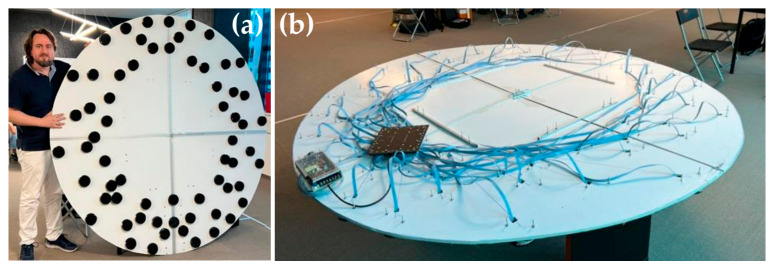
AC mounted on a cardboard circle for testing purposes: (**a**) front side with microphones; (**b**) back side with central board and a test power supply.

The front and the back side of the cardboard-mounted AC are depicted on the left and right panels of Figure 9, respectively. Cardboard and power supply included, the AC weighs 18 kg. Attached directly to the UAV frame and powered by the UAV PS, the AC will weigh less than 15 kg, which is an acceptable load for an airship that is dedicated to carrying sensors and instruments.

The AC consumes 40 W of power during regular operation, being powered with a 5 V source and drawing 8 A of current. However, the inrush current is somewhat higher. Initial tests indicate that the inrush current during power-on reaches approximately 12 A. After the transition period, when all capacitors are fully charged, power consumption reaches a steady state.

### 3.2. Acoustic Measurements

**Figure 10:**
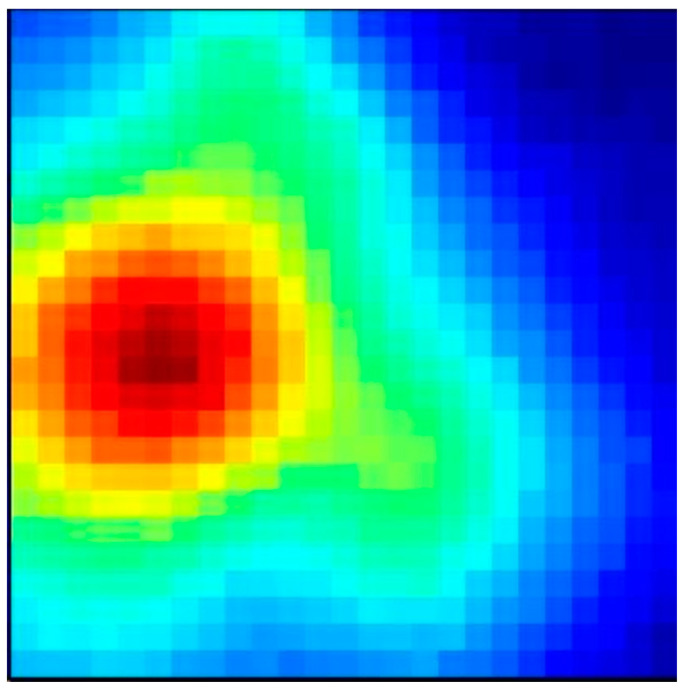
An indoor acoustic map with 25 × 25 pixels.

An acoustic map obtained with the AC is depicted in Figure 10. The resolution of 25 × 25 pixels for an image that covers a ground area of 300 m × 300 m implies a spatial resolution of 12 m. The map shown in Figure 10 depicts an event during which a person stood 3 m away from the camera indoors and said the word “sound” in a normal voice. A video recording of the event is available on a public repository [26]. The sound of the person speaking is clearly noticeable on the map, coming from a dominant direction.

**Figure 11:**
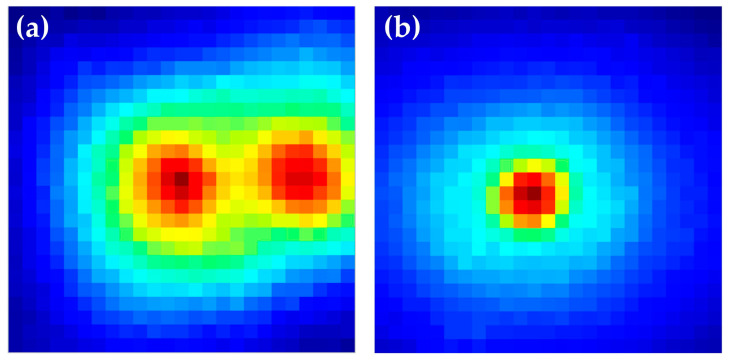
Acoustic maps of sources of pink noise recorded in outdoor conditions. (**a**) Two sources 3 m apart, 15 m away from the AC; (**b**) a single source in the middle of the field of view of the AC.

We also tested the outdoor operation of the camera. On the same circular board, the camera was brought outside and fixed upright so that it records in the forward direction at ground level. Two volunteers stood in front of the camera, 15 m away from it, and played pink noise from their handheld devices. Figure 11 depicts the acoustic maps obtained during those measurements. Figure 11a depicts two sources of pink noise, 15 m away from the sensor and 3 m apart from each other. Figure 11b depicts a single source of pink noise in the middle of the field of view of the sensor. It is expected that the operation of the AC will be little affected by the complex environment on a UAV, given that all components conform to industrial standards and that they are attached to the interior hull of the UAV, shielded from wind and other elements by the external UAV shell.

**Figure 12:**
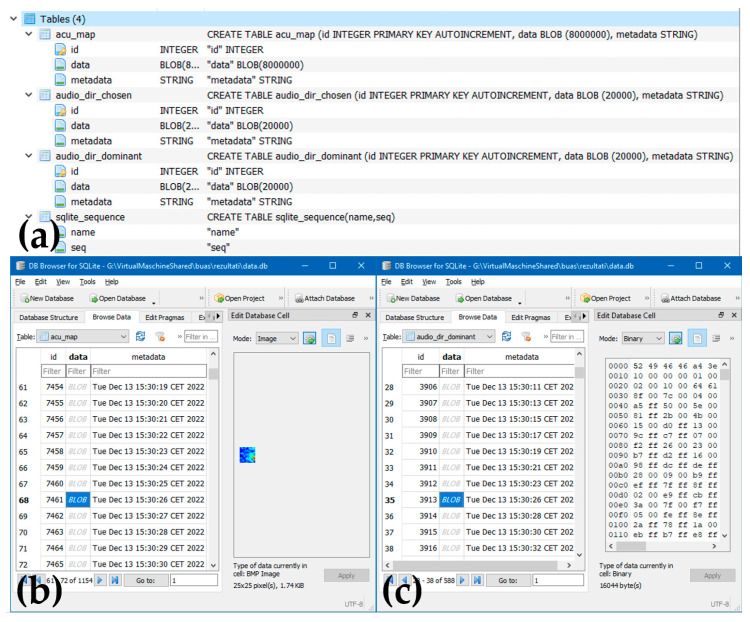
The AC SQLite database storing data during operation, shown with DB Browser for SQLite: (**a**) list of tables; (**b**) acoustic map table (bmp data in the BLOB field); (**c**) dominant direction table (wav data in the BLOB field).

An example of the data stored in the database during operation is depicted in Figure 12. For testing on the ground and operational demonstration, the communication software was run on an external Linux device that was connected to the AC. For UAV operation, the software will be installed on the UAV main board, and the data will also be stored on the UAV board. An exemplary database is also provided as part of the Supplementary Materials. The provided database can be used as inspiration by researchers who are constructing their own ACs, by professors teaching acoustics to their students, or for further numerical analysis.

## 4. Discussion

We have demonstrated prototype operation of an AC that is designed for mounting onto the hull of an airship. Mounting an AC onto an airship allows aerial sound surveillance for a number of applications, including urban and industrial noise monitoring, rescue missions, border surveillance, and wildlife monitoring. The AC has an aperture of two meters and has been designed for surveillance from a height up to 300 m.

The spatial resolution of the AC, indicated to be 12 m in the discussion of Figure 11, can be increased by improving the efficiency of the implementation of the beamforming algorithm on the PL of the CPU. A review of the available on-board resources indicate that it is possible to increase sampling frequency to at least 16 kHz, which is planned to be conducted in future work. Utilizing all the on-board resources would increase the spatial resolution to 6 m for the height of 300 m.

In real UAV-based operation, the absolute position of the dominant sound source can be determined and calculated from the GPS information provided by the UAV (longitude, latitude, and altitude) and from the UAV vertical axis tilt. On a UAV that contains multiple sensors, the acoustic map can be overlapped with images from other sensors (IR camera, high-resolution optical sensor, LIDAR, etc.).

UAV propulsion is a source of significant noise. Propeller noise would mask any useful acoustic signal reaching the microphones; thus, it is required to attenuate this signal enough to successfully extract the useful signal. Some experiments were conducted with a linear predictor based on the LMS algorithm [27] applied in sub-band adaptive filtering for drone propeller noise cancelation. Although promising results were obtained in terms of noise cancelation (the algorithm converged, with noise cancellation of approximately 30 dB with pre-selected segments of the recordings), it is still a small level of noise cancelation for such a low-level useful signal (up to 30 dB). In addition, the filtering of the useful part of the signal was not satisfactory. Therefore, application in UAVs will require the propulsion system to be stopped during acoustic acquisition. In addition, the inherently small signal-to-noise ratio (SNR) could be improved by applying deep-learning methods such as those used for low-light image and video enhancement [28]. Other limitations include spatial resolution which is set by the largest dimension of the camera, which in turn is limited by the size of the airship. For modern airships with dimensions on the order of 10’s of meters, flying at altitudes of 100’s of meters, the maximum ground resolution of ACs is on the order of several meters.

Future work will be oriented towards mounting the camera onto the UAV, recording during flight, algorithm updates to account for environmental noise, and integrating with the general UAV interface that may be designed for a multitude of sensors.

## Data Availability

The data presented in this study are openly available in Mendeley Data at doi.org/10.17632/WK8ND5ZJYF.1 [26].

## Supplementary Materials

The following supporting information can be downloaded at: https://www.mdpi.com/article/10.3390/s23020880/s1, Figure S1: The carbon construction tubes of the UAV with the positions of the microphones and the main board indicated; Figure S2: The microphone case with a wind shield and EMI shielding. Database of a recording: acu_data.db.
