# Supplementary material for: An Acoustic Camera for Use on UAVs
Source: Sensors (Basel). 2023 Jan 12;23(2):880. doi: 10.3390/s23020880 (PMC9865301; doi:10.3390/s23020880)
Supplement: Supplementary file 1 [file sensors-23-00880-s001.zip › sensors-2133165-supplementary.pdf]

An Acoustic Camera for use on UAVs

Iva Salom, Goran Dimić, Vladimir Čelebić, Marko Spasenović, Borislav Budisavljević, Milica Raičković, Mirjana Mihajlović, Tamara Vuković, Dejan Todorović

Supplementary Information

Optimal positions for the microphones on the carbon tube construction of the UAV hull are depicted in Figure S1. The green circles marked by red numbers are positions of the individual microphones. The position of the main board is marked with a green square. Figure S1 represents a top view of the internal construction of the UAV (Hipersfera Ltd., Zagreb, Croatia).

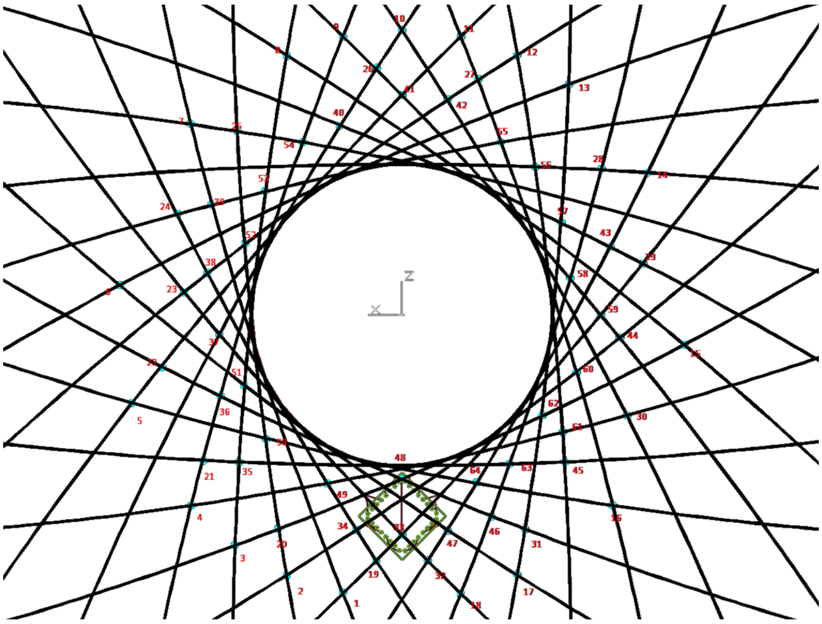

Figure S1: The carbon construction tubes of the UAV with the positions of the microphones and the main board indicated.

A microphone encapsulated in wind and EMI shielding in depicted in Figure S2.

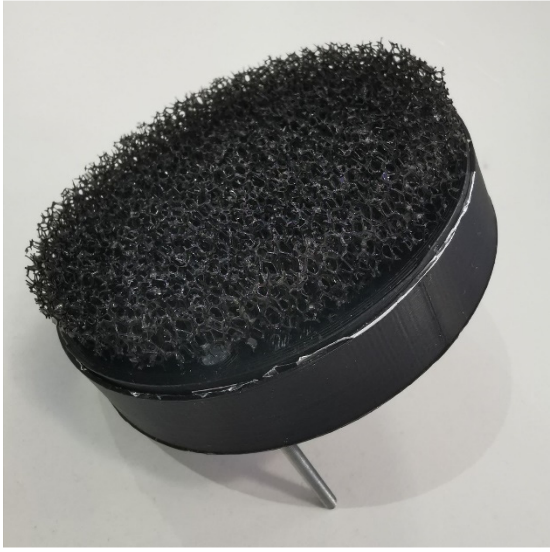

Figure S2: The microphone case with a wind shield and EMI shielding (aluminium foil glued on the inside faces the case).
